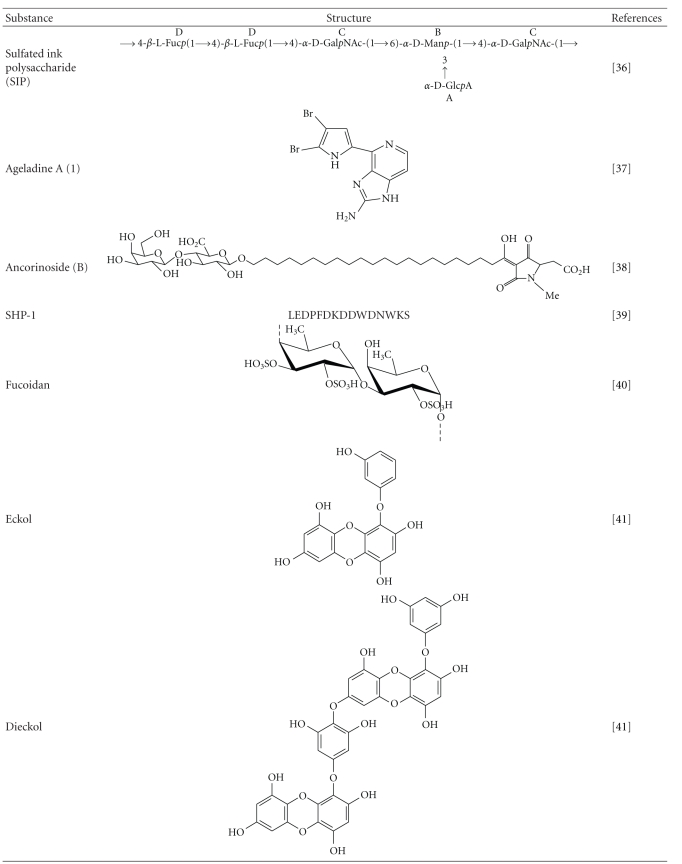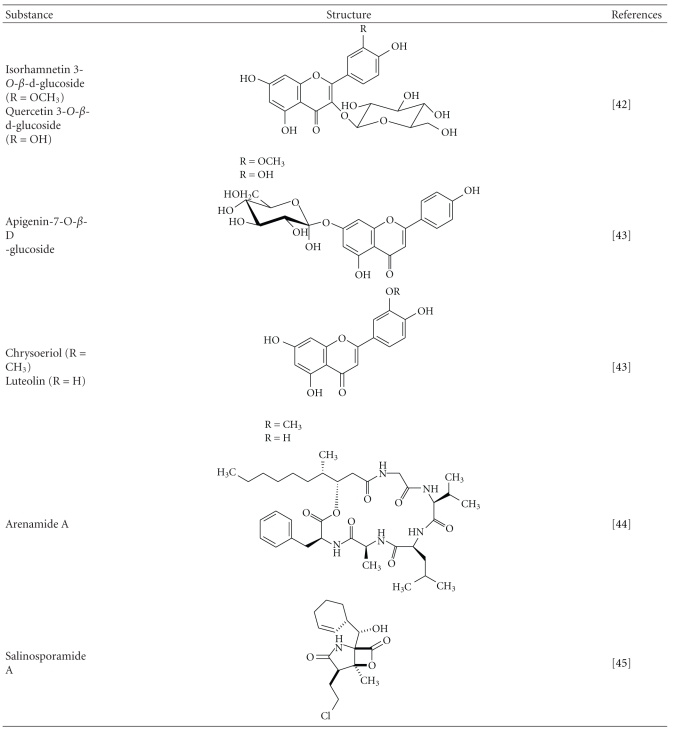# Metalloproteinase Inhibitors: Status and Scope from Marine Organisms

**DOI:** 10.1155/2010/845975

**Published:** 2010-12-09

**Authors:** Noel Vinay Thomas, Se-Kwon Kim

**Affiliations:** ^1^Marine Biochemistry Laboratory, Department of Chemistry, Pukyong National University, Busan 608-737, Republic of Korea; ^2^Marine Bioprocess Research Center, Pukyong National University, Busan 608-737, Republic of Korea

## Abstract

Marine environment has been the source of diverse life forms that produce different biologically active compounds. Marine organisms are consistently contributing with unparalleled bioactive compounds that have profound applications in nutraceuticals, cosmeceuticals, and pharmaceuticals. In this process, screening of natural products from marine organisms that could potentially inhibit the expression of metalloproteinases has gained a huge popularity, which became a hot field of research in life sciences. Metalloproteinases, especially, matrix metalloproteinases (MMPs) are a class of structurally similar enzymes that contribute to the extracellular matrix degradation and play major role in normal and pathological tissue remodeling. Imbalance in the expression of MMPs leads to severe pathological condition that could initiate cardiac, cartilage, and cancer-related diseases. Three decades of endeavor for designing potent matrix metalloproteinase inhibitory substances (MMPIs) with many not making upto final clinical trials seek new resources for devising MMPIs. Umpteen number of medicinally valuable compounds being reported from marine organisms, which encourage current researchers to screen potent MMPIs from marine organisms. In this paper, we have made an attempt to report the metalloproteinase inhibiting substances from various marine organisms.

## 1. Introduction

Metalloproteinases are a family of proteolytic enzymes generally termed as endopeptidases. This group of endopeptidases majorly consists of enzymes from metzincin family that include serralysins, astacins, adamalysins (a disintegrin and metalloproteinase domain or ADAMs), and matrixins (matrix metalloproteinases or MMPs) [1, 2]. The involvement of the regulated degradation of extracellular matrix (ECM) is essential for the physiological remodeling processes like tissue repair, development, and morphogenesis. Interestingly, the remodeling process found to be uncontrolled and deleterious immunological response to repair the tissue damages, which was credited by cardio-related ailments, cancer, and arthritis [3]. MMPs are exceptionally studied and focused because of their evident role in carcinogenesis and cellular invasion by catabolising the ECM [2]. MMPs are zinc-dependant endopeptidases that degrade the ECM [4] and this remodeling of ECM facilitates several physiological processes like wound healing, bone resorption, uterine involution, and organogenesis as well as pathologic conditions including inflammatory, vascular, and autoimmune disorders, and carcinogenesis [5]. Until now, about 25 MMPS have been reported in which 24 are found in mammals [6]. It was assumed that the MMPs and their role was confined to the degradation of ECM. However, recent scientific findings from several groups have established that MMPs cleave a wide range of extracellular, bioactive substrates, and regulating the activity of such proteins, typically in a gain-of-function manner, may indeed be the predominant function of MMPs *in vivo, *[6, 7]. In addition to that, MMPs play a predominant role in tumor invasion, angiogenesis, metastasis, transformation of cancer cells, signal transduction, and apoptosis [8]. 

Structurally, MMPs share a common domain structure, which includes propeptide domain, a catalytic metalloproteinase domain, a linker peptide often referred as hinge region and a hemopexin domain [9]. The catalytic zones of MMPs are compact, spherical and approximately 165 amino acid residues long with a substrate binding cleft. The extended zinc-binding motif harbors 3 zinc-binding histidines and a glutamate that preferentially serves as acid/base during catalytic reactions. MMP molecules possess three *α*-helices and a five-stranded *β* sheet as well as at least two calcium sites and a second zinc site which renders structural functions for these molecules [10]. The substrate specificity of MMPs is determined by a hydrophobic pocket called S1′ pocket which is found at the catalytic domain. Hence the S1′ pocket that determines the substrate specificity for MMPs becomes an inevitable source to devise MMP inhibitors [10]. 

Generally, intercellular regulation and cell matrix adhesion is regulated in a controlled manner, however, predominant human-related cancers are found with the dysregulation of these two phenomena. These pathological changes are due to the superior expressions of MMPs, the proteolytic enzymes [11]. The regulation of MMPs is controlled by endogenous inhibitors like tissue inhibitors of metalloproteinase (TIMPs), *α*_2_-macroglobin, heparin, and the reversion-inducing cysteine-rich protein with kazal motifs. TIMPs are naturally occurring inhibitors of MMPs and they form noncovalent 1 : 1 stoichiometric complexes with MMPs and prevent the proteolytic degradation [12]. Although the TIMPs have inhibitory activities on MMPs, they differ in many aspects like solubility, interaction with proenzymes (Pro-MMPs) and also regulation of expression [13]. Interactions and regulation of specific MMPs and other proteases by specific members of TIMP family are thoroughly discussed elsewhere [14–17]. 

MMPs association with various pathological responses has initiated an outstanding scientific preference to design the most potent MMPIs in the last 20 years. Many potential MMPIs have been formulated, however the challenging concept was that these inhibitors displayed poor selectivity towards MMP members and also the significance of blocking unrelated zinc proteases became an obstacle [18]. The strategies of designing MMPIs were based on the chelating of Zn^2+^ that induces inhibition of MMPs. Chelating groups like hydroxyl amine, carboxyl, thiol and so forth, were predominantly used. Commercially successful MMPIs like Batimastat (BB-94), Marimastat (BB-516) are known to have strong Zn^2+^ chelating group, hydroxamate. The poor selectivity, improper metabolism, low oral bioavailability, poor solubility, side effects like musculoskeletal pain and inflammation, complications and the risk of increased drug toxicity have strongly eliminated the first generation MMPIs from clinical trials [9, 19, 20]. 

As the above discussed scientific observations clearly bring front the point of reported MMPIs' failure in clinical trials, the need of designing novel MMPIs specific to MMPs is being the major concern. The new generation MMPI research suggests that the thorough study on S1′ pocket of MMPs could increase the selectivity of MMPs and also is thought to prove beneficial in reducing the risk of other MMP-related diseases [21, 22]. According to Chen et al., the MMPIs can be broadly divided into 4 classes: the natural MMPIs secreted by tissues; synthetic MMPIs; MMPIs screened from natural products; the MMPIs screened from the phage display random peptide library and antibody library. However, as the synthetic MMPIs indeed have few shortcomings at clinical usage, MMPIs derived from natural sources are being considered more for current time research. Until now, umpteen number of research groups have worked to achieve MMPIs from terrestrial sources and reported several outstanding natural MMPIs [23, 24]. For ages, it has been believed and proved that oceans harbor a variety of life forms ranging from microorganisms to vertebrates, which in turn provide mankind with several benefits biologically and medicinally. This feature of wide diversity in marine life forms has been identified as chief source for unique biologically active compounds that exhibit tremendous potential for pharmaceutical applications [25]. As these oceanic organisms are not completely exploited or studied well for their medicinal values, this field of research has gained lots of attention these days. This paper narrates a descriptive past research work carried out on MMPIs derived from marine organisms and in addition, the specific areas of metalloproteinase research have been outlined in a perspective manner.

## 2. Marine Animals

Fish from marine sources are abundant in omega-3 long-chain polyunsaturated fatty acids (*ω*3 LC-PUFAs) like eicosapentaenoic acid (EPA) and docosahexaenoic acid (DHA). Judé et al. have reported and compared some proposed mechanisms for the involvement of omega-3 LC-PUFAs in both cardiac and breast cancer protection [26]. Simple products like dietary oils from fish have been reported to exert beneficial properties in lowering the expressions of proMMP-2 and proMMP-9, that makes the dietary fish oil, a potential MMP inhibiting substance and can reduce the risk of inflammatory joint diseases [27]. Another research group has reported that oleic acid (OA), docosahexaenoic acid (DHA), and eicosapentaenoic acid (EPA) have significantly inhibited the lung metastasis by colon-carcinoma-26 cells by downregulating the activities of MMP-2 and -9 [28]. Diets supplemented with DHA reduce the metalloproteinase production in uterus, placenta, and liver tissues of rat. These findings suggest that competitive incorporation of *ω*3 LC-PUFAs and arachidonic acid into membrane phospholipids would consequently affect the MMP activities by bringing a change in the production of prostaglandin PGE2 [29]. A 21-kDa proteinase inhibitor from *Gadus morhua* (Atlantic cod) that shares similar properties to that of human TIMP-2 was investigated by Lødemel et al., whose studies revealed that this inhibitor has suppressed the gelatinolytic activity obtained from a human macrophage cell medium rich in MMP-9 [30], recommending it as a potent MMP inhibiting marine substance. Similar to this, shark cartilage is a source of antiangiogenic and antitumor compounds [31]. A novel antiangiogenic and antiinflammatory agent, AE -941 from shark cartilage has been studied by Dupont et al., who reported the effectiveness of this substance in treating psoriasis [32]. Compounds extracted from shark cartilage such as Neovastat, AE-941, U-995 have been checked for their antiangiogenic and antimetastatic effects. Neovastat has been found to inhibit MMP-2 and partially MMP-1, -7, -9 and -13.Western blot analysis confirms the presence of TIMP-like proteins within AE-941 that might be responsible in inhibiting the MMPs [33]. Our previous studies of potent ACE inhibitory peptide from tuna frame protein (PTFP) hydrolysate has shown antihypertensive effect in spontaneously hypertensive rats (SHRs) [34]. Earlier scientific investigations proved that ACE and MMPs are closely related to coronary diseases and the ACE inhibitors not only target ACE but also MMPs. Yamamoto and Takai have postulated that molecularinteraction between MMP-9 active sites and ACE inhibitors could brighten up the chances for MMP inhibitors for cardioprotection [35]. 

Marine cephalopods and their metabolites have been reported to have many applications in the field of medicine. SIP-SII is the sulfated ink polysaccharide (SIP) isolated from cuttlefish; *Sepiella maindroni *has been evaluated for its ability to inhibit the invasion and migration of SKOV3 and ECV304 cells via inhibition of MMP-2 proteolytic activity [36]. The backbone of SIP-SII is composed of fucose, *N*-acetylgalactosamine, mannose, and a single branch of glucuronic acid at the C-3 position of mannose with 34.7% of sulfated group [46]. Sulphated polysaccharides are known for their inhibition potential of MMPs [47–50] and especially the metastasis in cancer is strongly inhibited by the *O*-Sulfated polysaccharide [51]. Thus SIP-SII can be considered as a substance that can inhibit MMP-2 expression. According to Chen et al., SIP-named TBA-1 from the ink of *Ommastrephes bartrami *potentially inhibits the cell invasion and migration in HepG2, which proved once again that SIPs are becoming the best candidates as metalloproteinase inhibiting substances [52]. 

Marine sponges by being the most diversified faunal communities of seas, have contributed in discovering potential MMP inhibiting compounds. Ageladine A (**1**), a fluorescent alkaloid isolated from marine sponge *Agelas nakamurai *has the capability of inhibiting not only MMP-2 but also MMP-1, -8, -9, -12, and -13. This compound also inhibited the migration of bovine aortic endothelial (BAE) cells significantly. However, the mechanism of inhibiting the MMPs is not clear because the research results say that Ageladine A (**1**) did not chelate the Zn^+2^ and moreover the mode of inhibition is presumed to be other than the competitive inhibition [37]. Another study on Ancorinosides B, C, D isolated from the marine sponge *Penares sollasi* Thiele has shown an inhibitory effect on membrane type 1 matrix metalloproteinase (MT1-MMP) with IC_50_ values of 180–500 *μ*g/mL. These ancorinosides contain two carboxylic acids and a tetramic acid group and it is presumed that the latter plays an effective role in the inhibition of MMPs [38]. An antibacterial brominated compound produced by certain sponges named (+)-aeroplysinin-1 exhibited a clear inhibitory effect on the migration capabilities of endothelial cells. The inhibitory effects on MMP-2 and urokinase are revealed by the zymographic studies suggesting aeroplysinin-1 as a promising drug for angiogenesis-related pathologies [53]. According to the review by R. G. Kerr and S. S. Kerr debromohymenialdisine and its related compounds from a sponge genus *Hymeniacidon* were reported to inhibit the calf joint cartilage degradation by downregulating the metalloproteinases [54]. *Poecillastra* sp. and *Jaspis* sp. are known to harbor Psammaplin A (PsA) a natural bromotyrosine [55], where PsA is reported to have strong antiproliferative, antiangiogenic properties, which undoubtedly suggest PsA as a potential antiangiogenic agents [56]. 

Recently, a novel oligopeptide SHP-1, rich in acidic amino acids, aspartic acid (Asp) and glutamic acid (Glu) was isolated from sea horse *Hippocampus Syngnathidae *[39]. The sequence of the isolated oligopeptide is LEDPFDKDDWDNWKS, which was investigated for its ability to inhibit the expression of MMP-1, -3 and -13 thus preventing the cartilage damage in human chondrocytic (SW-1353) and osteoblastic (MG-63) cells. These results strongly support that metalloproteinases possess binding sites that are open for Asp and Glu and also Asp helps in rejuvenation of cell formation, cellular activity, and metabolism [57]. The cartilage preventive property of SPH-1 could be possible because of its down regulatory effect on the expression of MMP-1 and -13 which are thought to play a key role in the destruction of cartilage [58]. The same research group has reported pronase E-derived hydrolysate peptide of 1821 Da from a sea horse,* Hippocampus kuda* Bleeler.This peptide has been checked for its induction of differentiation of osteoblastic MG-63 and chondrocytic SW-1353 cells by measuring ALP activity, mineralization, and collagen synthesis. Interestingly, their results indicate that the respective peptide has considerably inhibited the MMP-1, -3 and -13 by picking the mechanism of reducing p38 kinase/NF-*κ*B in MG-63 cells and MAPKs/NF-*κ*B in SW-1353 cells [59]. These results clearly indicate that marine organisms are an excellent choice to devise MMPIs. Analysis of a gene for naturally occurring TIMP from pacific oyster *Crassostrea gigas *named cg-TIMP, the first tissue inhibitor of metalloproteinase from mollusks, revealed that it is closely related to vertebrate TIMP family and is effectively involved in wound healing as well as in defense mechanisms [60]. Exploitation of this feature by the use of gene technology could result in potential natural MMPI mimics that could be compatible for humans. 

Mucopolysaccharide called chitin from crustacean shells has been known for its vast application in nutraceuticals and as well as pharmaceuticals. Kong et al. synthesized water-soluble derivatives of chitin and chitosan, carboxymethyl-chitosan (CM-chitosan) and carboxymethyl-chitin (CM-chitin) by means of carboxymethylation reaction and evaluated their effect on the expressions of MMPs. As per their investigations, CM-chitin exhibited a higher MMP inhibitory, (MMP-2 and MMP-9) effect than CM-chitosan through transcriptional downregulations of activator protein-1 (AP-1) and nuclear factor *κ*B (NF-*κ*B), thus reporting CM-chitin and CM-chitosan as strong antioxidants and potent MMP inhibiting natural polymers [61]. Chitooligosaccharides (COS) ranging from 3–5 kDa has been reported to have a strong inhibitory effect on MMP-2 by decreasing the gene expression and transcriptional activity of MMP-2. Moreover COS of the same molecular weight represses the gene expression of c-fos, a part of AP-1 transcription factor suggesting that they can combat metalloproteinase-related pathological processes like metastasis and wrinkle formation [62]. Rajapakse et al. have reported that carboxylated chitooligosaccharides (CCOS) downregulate the MMP-9 expression in human fibrosarcoma (HT-1080) cells. In addition, CCOS is able to reduce the expression of MMP-9 by downregulating the MMP-9 at transcription level, mediated via the inhibition of AP-1 [63]. 

Brito et al. have reported about antiinflammatory properties of a heparin-like compound from the shrimp *Litopenaeus vannamei. *Their observations postulated that this compound has not only reduced the influx of inflammatory cells to injury site but also has an outstanding capability of inhibiting MMP-9 activity to 90%. The structural feature of this heparin-like protein from shrimp which has high content of glucuronic acid and disulfated disaccharide units renders the anticoagulant and hemorrhagic activities, which clearly recommends this heparin like substance as better antiinflammatory drug [64]. Heparinoid from shrimp has been reported to have potent antiangiogenic and antiinflammatory activities with insignificant anticoagulant or hemorrhagic actions, thus making shrimp heparinoid as a potent drug to treat neovascular age-related macular degeneration (AMD) and other angioproliferative diseases [65].

## 3. Marine Plants

Metabolites from marine plants have been reported to have outstanding biological activities. A highly effective anticoagulant and antiproliferative agent called, sulfated polysaccharide has been reported from a brown alga *Ecklonia cava*, which exhibited a promising antiproliferative effect on human promyelocytic leukemia (HL-60) and human leukemic monocyte lymphoma (U-937) cells at an IC_50_ value of 43.9 *μ*g/mL [66]. Fucoidan extracts from seaweed *Cladosiphon novae-caledoniae* Kylin (Mozuku) have reduced the cellular invasiveness in human fibrosarcoma HT1080 cells by suppressing the activity of MMP-2 and MMP-9. Further, it has been reported that these fucoidan extracts suppressed the expression and secretion of an angiogenesis factor, vascular endothelial growth factor (VEGF) thereby reporting the inhibitory effects on invasion and angiogenesis of tumor cells [67]. Fucoidan from *Costaria costata *of the order Laminariales, has an outstanding inhibitory effect of minimizing UV-B-induced MMP-1 expression by 41.8% at 0.01 *μ*g/mL, 57.7% at 0.1 *μ*g/mL, and 70% at 1 *μ*g/mL, compared to UVB irradiation alone (*P* < .05) in immortalized human keratinocyte (HaCaT) cells [40]. 

Methanolic extracts from marine red alga *Corallina pilulifera *(CPM) have potentially reduced the expression of UV-induced MMP-2 and MMP-9 in human dermal fibroblast (HDF) cell suggesting CPM as an effective antiphoto ageing agent [68]. In spirit of designing new antiphotoaging agents, Joe et al. reported that extracts from 3 species of Alariaceae, *Eisenia bicyclis, Ecklonia cava,* and *Ecklonia stolonifera*, have strongly reduced MMP-1 expression via inhibiting both NF-kappaB and AP-1 reporter. Moreover attenuation of MMP-1 expression was evident by treatment with eckol or dieckol, which were purely isolated from *E. stolonifera* in human dermal fibroblasts [41]. The phlorotannins eckol and dieckol were first isolated from *Ecklonia* species possess oligomeric polyphenol of phloroglucinol unit [69]. Various biological activities including free radical scavenging activity of phlorotannins from *Ecklonia* species have been reported earlier [70]. Dieckol from marine brown alga, *E. cava *has been reported to suppress LPS-induced production of nitric oxide (NO) and prostaglandin E_2_ (PGE_2_) and expression of inducible nitric oxide synthase (iNOS) and cyclooxygenase-2 (COX-2) in murine BV2 microglia, thus establishing dieckol as a potent antiinflammatory and neuroprotective agent [71]. 

*Salicornia herbacea*, a halophyte that grows on salt marshes and muddy seashores along the western coast of Korea. Kong et al. have reported that flavonoid glycosides, isorhamnetin 3-*O*-*β*-d-glucoside, and quercetin 3-*O*-*β*-d-glucoside isolated from this plant have inhibited the expression of MMP-2 and MMP-9 and elevated the TIMP-1 expression in human fibrosarcoma (HT1080) cells, which was confirmed by western blot. The biological activity of these flavonoids is believed to be contributed by the presence of phenolic hydroxyl groups [72]. Moreover, the downregulation of MMP-9 and MMP-2 by these flavonoids is due to the interference with the transcription factor AP-1 there by suggesting these flavonoid glycosides as potent natural chemopreventive agents for cancer [42]. Mook-Jung et al. have worked on the constituents of two *Rhodiola* plants, *Rhodiola sacra* S. H. Fu and *Rhodiola sachalinensis* A. Bor, reporting that out of the 58 compounds tested, six had considerable protective effects against beta-amyloid-induced death of B103 neuronal cells *in vitro* [73]. The extracts from sea grass *Zostera marina L.*, apigenin-7-O-*β*-D-glucoside (1), chrysoeriol (2), and luteolin (3) can scavenge reactive oxygen species. Especially, luteolin has remarkable inhibitory activity on MMP-1 for upto 44% at 4.0 uM. The research results suggest that luteolin (3) inhibited the MMP-1 expression by suppressing the IL-6 expression by 30% at 4.0 *μ*M [43]. It suggests that these extracts can combat photoaging induced by UV rays.

In the exploration of marine natural antioxidants, two bioactive isolates floridoside and d-isofloridoside from marine edible red alga *Laurencia undulata* have been studied by Li et al. and are reported for their profound effect on scavenging free radicals and inhibiting the metalloproteinases MMP-2 and -9. The structure-activity relationships (SARs) studies suggest that the effective antioxidant activity of these floridosides is rendered by the galactose group and glycerol residue which can donate hydrogen ion and in turn excited hydroxyl groups can attract electrons easily [74]. Phlorotannins from brown alga *E. cava* have been reported to have inhibitory activity on MMP-2 and MMP-9 signifying the role of phlorotannins as potential and safe marine derived MMPIs [75]. These findings clearly suggest that purified extracts from *E. cava *inhibit MMP activity at a lower concentration and reduce the incidence of metastasis and skin wrinkle *in vivo* model.

## 4. Marine Microorganisms

Around 70%–80% of marine environment is dominated by various kinds of microorganisms that possess biologically active compounds. Calcium spirulan (Ca-SP) isolated from *Spirulina platensis*, a marine unicellular alga was able to reduce the melanoma (as studied in B16-BL6 cells), carcinoma (evidenced in colon 26-M3.1 cells), and fibrosarcoma (evidenced in HT-1080 cells). These findings confirm the possible role of Ca-Sp in interfering with the MMPs and disabling the tumor invasion [76]. Yang et al. reported that polysaccharide extract from *S. platensis* (PSP) inhibits corneal neovascularization (CNV) caused by alkali burns. Their findings clearly show that application of PSP has reduced several parameters related to CNV and/or inflammation including the downregulation of MMP-2 and MMP-9 [77]. Species of *Chlorella* are known for their vast application in nutraceuticals. However, antioxidants extracted from *Chlorella vulgaris *by a special supercritical carbon dioxide extraction (SC-CO_2_) technology has proven advantage in inhibiting MMP-mediated cancer proliferation and progression via the suppression of cellular invasion and metastasis [78]. 

Asolkar et al. reported arenamides A-C (**1**–**3**), from *Salinispora arenicola*, a marine bacterial strain and the effect of arenamides A and B on NF*κ*B activity in stably transfected to 293/NF*κ*B-Luc human embryonic kidney cells, which revealed an outstanding blockage of  TNF-induced activation. As expression of MMP-9 gene is an outcome of NF*κ*B activity, further sophisticated studies could make the arenamides potential MMP inhibiting compounds [44]. Marine bacterium *Salinispora tropica *renders salinosporamide A (also called NPI-0052), which was recently identified by Ahn et al. They have monitored the salinosporamide A's ability to potentiate the apoptosis induced by tumor necrosis factor-*α* (TNF-*α*), bortezomib, and thalidomide, and this mechanism was correlated with the downregulation of cancer-related gene products including MMP-9 that plays a role in tumor invasion. Thus by suppressing the NF-*κ*B pathway salinosporamide proved to be a promising MMP inhibiting molecule [45].

## 5. Conclusions

In recent years, oceans' flora and fauna have been studied extensively for their biochemical responses to evade potential threats they encounter in marine locales. This has led to the idea of exploiting those biochemicals, in other words natural products to be used as potential inputs in medicinal applications that would prove to be beneficial in uplifting human health. The recent scientific investigations unleashing the prominence of metalloproteinases in several human-related ailments and the drawbacks of the currently available synthetic antimetalloproteinases encourages the present day researchers to explore natural resources for more devising reliable and specific MMPIs. Synthetic MMPIs have few shortcomings like nonspecific selectivity, improper metabolization that often leads to undesirable side effects, poor bioavailability and so forth. These have been the key reasons to put most of the synthetic MMPIs out of clinical trials. While on the other side, MMPIs from marine organisms like fish, mollusks, cephalopods, red algae, brown algae, few marine bacteria have reportedly given much promising results (Table 1). However, screening of MMPIs from marine organisms that specifically bind to the S1′ pocket of metalloproteinases and the substances which can show competence to bind with the active site of metalloproteinases is much challenging. Considering the species and technologies explored, it can be said that exploration has just confined to the tip of an ice berg. With the current cutting edge scientific approaches, more marine organisms and their products can be explored and studied for coming up with the apt marine natural MMPIs.

## Figures and Tables

**Table 1:** List of important substances from marine organisms that inhibit metalloproteinases.